# Selenium Biofortification in *Fragaria × ananassa*: Implications on Strawberry Fruits Quality, Content of Bioactive Health Beneficial Compounds and Metabolomic Profile

**DOI:** 10.3389/fpls.2017.01887

**Published:** 2017-11-06

**Authors:** Tanja Mimmo, Raphael Tiziani, Fabio Valentinuzzi, Luigi Lucini, Carlo Nicoletto, Paolo Sambo, Matteo Scampicchio, Youry Pii, Stefano Cesco

**Affiliations:** ^1^Faculty of Science and Technology, Free University of Bozen-Bolzano, Bolzano, Italy; ^2^Institute of Environmental and Agricultural Chemistry, Università Cattolica del Sacro Cuore, Piacenza, Italy; ^3^Department of Agriculture, Food, Natural Resources, Animals and Environment, University of Padova, Padova, Italy

**Keywords:** Se biofortification, strawberry, metabolomics/metabolite profiling, fruit quality, flavonoids, phenolic compounds

## Abstract

Selenium (Se) is an essential nutrient for humans, due to its antioxidant properties, whereas, to date, its essentiality to plants still remains to be demonstrated. Nevertheless, if added to the cultivation substrate, plants growth resulted enhanced. However, the concentration of Se in agricultural soils is very variable, ranging from 0.01 mg kg^-1^ up to 10 mg kg^-1^ in seleniferous areas. Therefore several studies have been performed aimed at bio-fortifying crops with Se and the approaches exploited were mainly based on the application of Se fertilizers. The aim of the present research was to assess the biofortification potential of Se in hydroponically grown strawberry fruits and its effects on qualitative parameters and nutraceutical compounds. The supplementation with Se did not negatively affect the growth and the yield of strawberries, and induced an accumulation of Se in fruits. Furthermore, the metabolomic analyses highlighted an increase in flavonoid and polyphenol compounds, which contributes to the organoleptic features and antioxidant capacity of fruits; in addition, an increase in the fruits sweetness also was detected in biofortified strawberries. In conclusion, based on our observations, strawberry plants seem a good target for Se biofortification, thus allowing the increase in the human intake of this essential micronutrient.

## Introduction

In most soils, selenium (Se) occurs in relatively low concentrations, ranging from 0.01 to 2 mg kg^-1^ (Marschner, 2011), although concentrations higher than 10 mg kg^-1^ can be found in seleniferous areas (Fordyce, 2007). Even though Se is an essential nutrient for humans and animals, its essentiality for higher plants still remains to be demonstrated. However, several studies have shown that, when Se was added to the substrate, the growth of both hyper-accumulator and non-hyper-accumulator plants increased (Hartikainen, 2005; Galeas et al., 2008). Furthermore, Se supplementation in both wheat and soybean has led to an enhanced resistance toward oxidative stresses and improved both mineral nutrients and vitamin E concentration (Kabata-Pendias, 2010).

Selenium is most likely absorbed by plants in the form of selenate (SeO_4_^2-^) and, due to its chemical and physical similarities, it competes with sulfate (SO_4_^2-^) for the same transport mechanisms at the root surface (Shibagaki et al., 2002; El Kassis et al., 2007). The uptake of selenate can thus be reduced by high concentrations of SO_4_^2-^ in the soil solution (Zayed and Terry, 1992), and vice versa; however, the levels of selenate in soils are usually far too low to affect sulfate uptake (approximately 20-fold lower) (Marschner, 2011). Selenium uptake capability strongly depends on the plant species, being the majority of the agricultural and horticultural plants classified as non-accumulators (Brown and Shrift, 1982). Nevertheless, it was shown that, among 37 non-accumulators species, there is a positive relationship between sulfur (S) and Se leaf concentration, showing that the uptake of selenate and sulfate is tightly linked (White et al., 2004). The main actor involved in the uptake of both anions is the high affinity SO_4_^2-^ transporter *Sultr1;2* (Barberon et al., 2008). In fact, in SO_4_^2-^ deficiency conditions, *Sultr1;2* was shown to be over-expressed in wheat plants, thus leading to an increased tissue concentration of selenate (Shinmachi et al., 2010).

Once taken up, Se is assimilated in plants *via* the sulfur assimilation pathway (Malagoli et al., 2015): selenite is activated by ATP sulfurylase and afterward reduced to selenide (Sors et al., 2005), which is then assimilated as modified amino acids (Kabata-Pendias, 2010). Despite being the antagonism between selenate and sulfate the most studied, Se has been shown to compete with other mineral nutrients for the uptake; for instance, molybdate and iodate are oxyanions that share some similarities with selenate and may use the same set of transporters to be taken up by plants (Shinmachi et al., 2010; DeTar et al., 2015); yet, Se is also known to impinge on the absorption of several metals, such as manganese (Mn), zinc (Zn), copper (Cu) and cadmium (Cd), as well as of the macronutrients nitrogen (N) and phosphorus (P) (Kabata-Pendias, 2010).

From a human and an animal nutritional point of view, Se is a key component of selenoproteins. Yet, the range between beneficial and harmful concentration of Se is relatively narrow. In fact the World Health Organization (WHO) and the United States Department of Agriculture (USDA) recommend a daily intake of 55–200 μg Se in regular adults (USDA, 2012) to reduce, for instance, the incidence of prostatic and lung cancer (Brummell et al., 2011; Dennert et al., 2011). Doses greater than 400 μg Se per day on the other hand, might produce toxic effects, resulting in pathological conditions (Rayman, 2008; Rayman, 2012). Considering its beneficial impact on the human health status, Se supplementation has been gaining importance in the last years. Plant-derived food represents thereby the main Se source for humans. Nevertheless, the Se concentration accumulated within plants strongly depends on several factors, as for instance the concentration of Se in soil, the concentration of competing S and the plant species itself (Malagoli et al., 2015). In recent years, several studies have been carried out aimed at bio-fortifying crops with Se (Chen et al., 2002; Lyons et al., 2004; Thavarajah et al., 2008; Schiavon et al., 2013; Hernández-Castro et al., 2015) and the approaches exploited were based on either the application of Se fertilizers, the genetic selection of Se accumulating plants or the genetic modification of crops to enhance Se uptake (Zhu et al., 2009). Nonetheless, considering the narrow gap between the beneficial and toxic concentrations of Se for human health, it is crucial to precisely tune the biofortification strategies of crops in order to optimize Se intake avoiding the accumulation of detrimental concentrations.

Strawberry (*Fragaria × ananassa*) is one of the most important and popular fruits with a great economical and commercial importance (Giampieri et al., 2012). Its great health benefit is represented by a very high content of micronutrients and phytochemicals (Tulipani et al., 2008; Giampieri et al., 2012). In fact, among fruits, strawberries have one of the greatest antioxidant activity (Halvorsen et al., 2002) with a folate content ranging from 20 to 25 μg 100 g^-1^ FW and an anthocyanin content ranging from 150 to 600 mg kg^-1^ FW (Castro et al., 2002). In addition, strawberries are one of the fruits with the highest macro- and micronutrient content, providing quiet high amounts of magnesium, phosphorus, potassium, manganese, iodine, copper and iron (Giampieri et al., 2012). Thus, strawberries and their derivatives might constitute excellent sources for nutraceutical compounds that can have a positive role in human health (Stoner and Wang, 2013). Recent evidence has pointed out that upon the variation of the composition of the growth medium, e.g., under limited Fe and P availability, fruits were richer in pelargonidin-3-glucoside, benzoic acids and flavonols (Valentinuzzi et al., 2015a). Likewise, also the Se fertilization has been shown in other plant species to alter the qualitative and quantitative compositions of nutraceuticals (Finley et al., 2005; Robbins et al., 2005; Ríos et al., 2008). To our knowledge, no study has been carried out to date with the aim of assessing the feasibility of Se fortification in strawberry and the implication of such practice on the nutritional value.

For these reasons, the aim of the present research was to assess the biofortification potential of Se in hydroponically grown strawberry fruits using three levels of Se. Furthermore, we also aimed at assessing the effect of Se enrichment in strawberry fruits on the qualitative parameters as organic acid and sugar content, titratable acidity, firmness and content in health-promoting compounds such as phenols, flavonoids and flavonols, as well as the metabolic changes induced by different Se levels. The hypothesis was thereby that Se biofortification might lead to a Se enrichment in strawberry fruits without worsening the fruit quality (due to an imbalanced S/Se uptake).

## Materials and Methods

### Plant Material and Growing Conditions

*Fragaria × ananassa* cv. Elsanta plants were hydroponically grown, as previously described (Valentinuzzi et al., 2015b), in either a full nutrient solution (control), composed as follows KH_2_PO_4_ 0.25 mM, Ca(NO_3_)_2_ 5 mM, MgSO_4_ 1.25 mM, K_2_SO_4_ 1.75 mM, KCl 0.25 mM, Fe(III)Na-EDTA 20 μM, H_3_BO_3_ 25 μM, MnSO_4_ 1.25 μM, ZnSO_4_ 1.5 μM, CuSO_4_ 0.5 μM, (NH_4_)_6_Mo_7_O_24_ 0.025 μM, or in a nutrient solution supplemented with 10 μM Se or 100 μM Se, supplied as Na_2_SeO_4_ (Sigma–Aldrich, reagent grade ≥ 98.0%). The nutrient solution was changed every 3–4 days. Plants were grown in a climate chamber with 14/10 h day/night, 24/19°C, 70% relative humidity (RH) and 250 μmol m^-2^ s^-1^ light intensity. Ten biological replicates were kept for each treatment.

### Plant Growth Parameters and Plant Analysis

Strawberry plants were hydroponically grown for 72 days. Strawberry fruits were harvested once at least 80% of the fruit surface showed a red coloration. Fruits skin color was evaluated determining the color parameters using a portable tristimulus colorimeter (Chroma Meter CR-400, Konica Minolta Corp., Osaka, Japan). Fresh weight (FW), yield per plant (g FW per plant), average fruit yield (g FW), average number of strawberry fruits per plant were assessed. At harvest, shoots and roots were separated assessing fresh weight (FW) and dry weight (DW) of the tissues together with the root to shoot ratios.

### Fruit Quality Assessment

Titratable acidity, total soluble solid content and firmness of fresh strawberry fruits were determined as previously described (Valentinuzzi et al., 2015a).

### Analyses of Strawberry Extracts

Freeze-dried strawberry samples were homogenized and 100 mg of strawberry powder were extracted with 1 mL methanol (HPLC grade, Merck, Darmstadt, Germany). The mixture underwent sonication for 30 min at 4°C and the extracts were centrifuged at 14000 × *g* for 30 min at 0°C; afterward, the supernatant was collected and filtered through a 0.2 μm nylon filter (Valentinuzzi et al., 2015a). The content of total phenols of strawberry fruit extracts was determined following the Folin-Ciocalteau method (Folin and Ciocalteu, 1927; Atanassova et al., 2011), whilst the concentration of flavonoids and flavonols was determined by a pharmacopeia method, using rutin hydrate as reference compound (Miliauskas et al., 2004).

### HPLC Analyses

Organic acids and sugars were separated simultaneously by HPLC using a cation exchange column Aminex 87-H column (300 × 7.8 mm, 9 μm, Bio-Rad) using an isocratic elution with 10 mM H_2_SO_4_ as carrier solution at a flow rate of 0.6 mL min^-1^. Organic acids were detected at 210 nm using a Waters 2998 photodiode array detector (Waters Spa, Italy), whilst sugars were detected by a refractive index detector (Waters Spa, Italy). Standard acids and sugars were prepared as individual stock solutions and then combined to give diluted reference standards. Organic anions and sugars were identified by comparing retention times of unknowns to pure compounds. Standards were purchased from Sigma–Aldrich (St. Louis, MO, United States).

### Elemental Analysis

Oven-dried shoot samples (60°C) and freeze-dried strawberry fruits were homogenized and approximately 0.5 g of each sample were acid digested with concentrated ultrapure HNO_3_ (650 ml L^-1^; Carlo Erba, Milano, Italy) using a single reaction chamber microwave digestion system (UltraWAVE, Milestone, Shelton, CT, United States). Selenium and macro- and micronutrient concentrations were then determined by ICP-OES (Arcos Ametek, Spectro, Germany), using tomato leaves (SRM 1573a) and spinach leaves (SRM 1547) as external certified reference material. The limits of detection for each element are reported as follow: Ca 1.80 μg L^-1^, Fe 0.32 μg L^-1^, K 1.10 μg L^-1^, Mg 1.53 μg L^-1^, Mn 0.37 μg L^-1^, P 2.00 μg L^-1^, S 9.00 μg L^-1^, Se 11.10 μg L^-1^.

### Metabolomic Analysis of Strawberry Fruits

The screening of plant metabolites was carried out on a hybrid quadrupole-time-of-flight mass spectrometer coupled to an UHPLC chromatographic system (UHPLC/Q-TOF) using a metabolomic analysis as previously set-up (Lucini et al., 2015). A 1290 liquid chromatograph, coupled to a G6550 Q-TOF mass spectrometer via a Dual Electrospray JetStream ionization system (all from Agilent technologies, Santa Clara, CA, United States), was used. Briefly, the instrument was run in the positive SCAN mode (MS-only) and operated to acquire spectra in the range of 100–1600 *m/z* in extended dynamic range mode. Reverse phase chromatographic separation was achieved using a Knauer BlueOrchid C18 column (100 × 2 mm i.d., 1.8 μm) and a gradient elution having as LC mobile phase a mixture consisted of (A) water (proteomic grade from VWR International, Radnor, PA, United States) and (B) methanol (LCMS grade from VWR). Formic acid 0.1% (v/v) and ammonium formate (5 mM) (both from Sigma–Aldrich, St. Louis, MO, United States) were added to both mobile phases. The gradient started with 5% B and increased to 90% B within 30 min, then was held for 5 min. The mobile phase temperature was set to 35°C, the injection volume was 3 μl and the flow rate was 220 μl min^-1^.

Lock masses were continuously infused via a dedicated electrospray in the JetStream source to ensure mass accuracy; purine at m/z 121.0509 and HP-0921 at m/z 922.0098 were used with this purpose.

The raw data gained from the Q-TOF mass analyzer were processed by the Profinder B.06 software (from Agilent Technologies) using the “find-by-formula” algorithm. Compounds identification was based on both accurate mass and isotopic pattern (i.e., accurate spacing and isotopes ratio), and expressed as overall identification score. Compounds were then aligned (mass and retention time), annotated using the database exported from PlantCyc 9.5 (Plant Metabolic Network^1^; released November 2014) and then subjected to a recursive analysis workflow having retention time as mandatory (with retention time tolerance of <0.1 min) in the second ID step. Once mass and retention time alignment was done, a filter-by-frequency post-processing filter was applied after deconvolution, retaining only those compounds that were present in at least 80% of replications within at least one treatment (Lucini et al., 2015). A second identification process, using the same procedure, was carried out to specifically profile phenolic compounds using the database Phenol-Explorer (Rothwell et al., 2013).

Therefore, based on the strategy applied, identification was carried out according to Level 2 (putatively annotated compounds) as set out by the COSMOS Metabolomics Standards Initiative^2^.

### Statistical Analyses

The results are presented as means of at least three replicates ± standard error (SE). All the quantitative analyses have been carried out on at least three biological independent replicates. In the case of fruit quality analyses, each single independent replicate was obtained by pooling ten strawberry fruits at the same ripening stage. Statistical analysis was performed using Statgraphics (Statpoint technologies, Inc., Warrenton, VA, United States). Data were analyzed by analysis of variance (ANOVA), and means were compared using Student Newman Keul’s (SNK) test at *p* ≤ 0.05 to determine the significance of differences found.

Interpretation of metabolomic results was carried out using Mass Profiler Professional B.12.06 (from Agilent technologies). Compounds abundance was log2 normalized, abundances were normalized at 75th percentile and baselined versus the median of each compound in all samples. For statistical analysis from metabolomic data, unpaired *t*-test (*P* ≤ 0.001, Bonferroni multiple testing correction) and fold-change analysis (cut-off = 2) were combined into volcano plots. Furthermore, a multivariate partial least squares discriminant analysis (PLS-DA, N-fold validation with *N* = 4) was performed and variables loadings, which were used to build the class prediction model, were plotted according to their weight within the latent vectors. Compounds with the highest scores on the first and second latent vectors were exported from the covariance structures (loading plot) in the PLS-DA hyperspace.

## Results

### Assessment of Plant Growth

Strawberry plants were hydroponically grown for approximately 10 weeks in either a full nutrient solution (control), or in a nutrient solution added with Se at a final concentration of 10 and 100 μM. Fruits were harvested once at least 80% of the fruit surface showed a red coloration, whilst shoots and roots were collected at the end of the growing period and subjected to subsequent analyses.

Shoots fresh weight increased by 20% in strawberries treated with 100 μM Se respect to the control plants and those treated with 10 μM Se (**Table 1**). Also the leaf area increased in strawberries treated with 100 μM Se by 17% in comparison with both control and 10 μM Se treated plants (**Table 1**). As a consequence and due to the fact that the root biomass did not increase with increasing Se concentration, the shoot to root ratio increased in plants supplied with the highest Se concentration (**Table 1**).

**Table 1:** Fresh weight, shoot to root ratio, leaf area, average yield per plant, average number of berries per plant and average berry weight of strawberries grown in a full nutrient (control) and a nutrient solution either supplied with 10 or 100 μM Se.

	Control	10 μM Se	100 μM Se	*P*
FW shoot (g per plant)	30.58 ± 2.65^b^	31.13 ± 3.56^b^	37.19 ± 3.57^a^	**0.021**
FW root (g per plant)	32.26 ± 1.77^ns^	32.26 ± 1.77^ns^	29.58 ± 1.22^ns^	0.186
Shoot/root ratio	0.94 ± 0.05^b^	1.13 ± 0.14^ab^	1.25 ± 0.09^a^	**0.027**
Leaf area (cm^2^)	41.98 ± 2.16^b^	41.52 ± 2.21^b^	49.51 ± 2.58^a^	**<0.001**
Average yield/plant (g)	43.84 ± 6.06^ns^	53.36 ± 7.94^ns^	53.06 ± 8.05^ns^	0.612
Average number of berries /plant	9.11 ± 1.08^ns^	10.90 ± 1.49^ns^	10.20 ± 1.50^ns^	0.670
Average berry weight (g)	4.79 ± 0.52^ns^	5.01 ± 0.53^ns^	5.14 ± 0.23^ns^	0.409

*FW, fresh weight; mean ± SE; letters following the means indicate significant differences; ns, not significant. Bold *p*-values highlight statistical significance.*

The chlorophyll content, evaluated as SPAD index values, was expressed as average between old and young leaves and it did not highlight any difference between the Se treated and control plants (**Supplementary Figure S1**).

### Mineral Nutrient Content

The micro- and macronutrient content has also been assessed in shoots and roots of strawberry plants to further study the effect of Se on plant physiology and possible nutrient imbalances (**Table 2**). In shoots, the Se concentration reached 10.48 ± 1.20 μg g^-1^ DW in plants supplied with 10 μM Se and 125.08 ± 13.89 μg g^-1^ DW in those supplied with 100 μM Se. Beside Se, only the concentration of S in the shoots of plants treated with 100 μM Se was significantly higher than in control and in 10 μM Se-treated strawberry plants (**Table 2**).

**Table 2:** Macro- and micronutrients concentration in strawberry shoots, roots and fruits grown in a full nutrient (control) and a nutrient solution either supplied with 10 or 100 μM Se.

	Control	10 μM Se	100 μM Se	*P*
**Shoot**				
P (mg g^-1^ DW)	3.65 ± 0.08^ns^	3.05 ± 0.54^ns^	3.60 ± 0.26^ns^	0.449
K (mg g^-1^ DW)	6.46 ± 0.20^ns^	5.84 ± 0.61^ns^	6.40 ± 0.01^ns^	0.432
Ca (mg g^-1^ DW)	15.03 ± 0.76^ns^	13.52 ± 2.30^ns^	14.01 ± 0.46^ns^	0.754
Mg (mg g^-1^ DW)	3.12 ± 0.09^ns^	3.05 ± 0.53^ns^	3.33 ± 0.05^ns^	0.813
S (mg g^-1^ DW)	1.55 ± 0.01^b^	1.54 ± 0.28^b^	2.26 ± 0.18^a^	**0.061**
Fe (μg g^-1^ DW)	87.81 ± 9.00^ns^	71.68 ± 9.86^ns^	79.10 ± 6.66^ns^	0.463
Mn (μg g^-1^ DW)	86.78 ± 5.96^ns^	73.20 ± 11.66^ns^	78.00 ± 15.69^ns^	0.724
Se (μg g^-1^ DW)	<LOD	10.48 ± 1.20^b^	125.08 ± 13.89^a^	**0.001**
**Root**				
P (mg g^-1^ DW)	2.86 ± 0.47^ns^	2.76 ± 0.21^ns^	3.04 ± 0.30^ns^	0.846
K (mg g^-1^ DW)	5.62 ± 0.22^b^	6.32 ± 0.04^a^	6.33 ± 0.06^a^	**0.004**
Ca (mg g^-1^ DW)	7.78 ± 0.37^b^	7.27 ± 0.26^b^	9.67 ± 0.71^a^	**0.011**
Mg (mg g^-1^ DW)	2.03 ± 0.35^b^	2.24 ± 0.13^b^	3.43 ± 0.28^a^	**0.007**
S (mg g^-1^ DW)	3.08 ± 0.37^ns^	3.32 ± 0.34^ns^	3.36 ± 0.25^ns^	0.808
Fe (μg g^-1^ DW)	1760.90 ± 167.41^ns^	1894.10 ± 242.20^ns^	1781.10 ± 124.70^ns^	0.861
Mn (μg g^-1^ DW)	32.80 ± 5.78^ab^	25.00 ± 3.10^b^	58.73 ± 15.21^a^	**0.067**
Se (μg g^-1^ DW)	<LOD	19.02 ± 2.99^b^	174.42 ± 14.35^a^	**<0.001**
**Fruits**				
P (mg g^-1^ DW)	3.43 ± 0.30^ns^	2.96 ± 0.04^ns^	2.83 ± 0.15^ns^	0.152
K (mg g^-1^ DW)	6.42 ± 0.05^ns^	6.42 ± 0.04^ns^	6.42 ± 0.03^ns^	0.999
Ca (mg g^-1^ DW)	2.56 ± 0.09^a^	2.09 ± 0.13^ab^	1.63 ± 0.20^b^	**0.012**
Mg (mg g^-1^ DW)	1.62 ± 0.13^ns^	1.45 ± 0.02^ns^	1.44 ± 0.06^ns^	0.312
S (mg g^-1^ DW)	1.06 ± 0.08^ns^	1.04 ± 0.02^ns^	1.05 ± 0.07^ns^	0.971
Fe (μg g^-1^ DW)	34.89 ± 3.59^ns^	30.86 ± 0.75^ns^	28.62 ± 1.34^ns^	0.217
Mn (μg g^-1^ DW)	27.10 ± 0.85^ns^	23.59 ± 1.13^ns^	23.58 ± 2.90^ns^	0.366
Se (μg g^-1^ DW)	<LOD	3.95 ± 0.40^b^	46.04 ± 4.37^a^	**0.002**

*DW, dry weight; LOD, limit of detection; mean ± SE; letters following the means indicate significant differences; ns, not significant. Bold *p*-values highlight statistical significance.*

In roots tissues, the Se concentration was about 19.02 ± 2.99 μg g^-1^ DW when supplied with 10 μM Se and about 174.42 ± 14.35 μg g^-1^ DW with 100 μM Se application (**Table 2**). Differently from shoots, the presence of increasing concentration of Se in the growth medium caused an alteration in the uptake of several macro- and micronutrients (**Table 2**). Calcium (Ca), magnesium (Mg) and manganese (Mn) showed higher concentration in roots in the case of 100 μM Se-treated plants, whilst potassium (K) concentration was increased in the roots of both 10 and 100 μM Se-treated strawberries (**Table 2**).

With the aim of determining whether the growth of plants in Se-fertilized hydroponic solution might result in the production of biofortified fruits, strawberries were analyzed for their macro- and micronutrient content (**Table 2**). In fruits, Se concentration showed increasing concentrations according with the increased Se supply to the nutrient solution. An addition of 10 and 100 μM Se led to an average Se content in fruits of 3.95 and 46.04 μg Se g^-1^ DW, respectively. It is well established that Se and S use the same transporters being taken up by plants, therefore the high availability of one of the two element might decrease the absorption of the other. Nevertheless, in our experimental conditions, no differences in the S concentration have been highlighted between treatments (**Table 2**). Likewise, except for Ca, any other element was altered significantly in Se-supplemented strawberries (**Table 2**).

### Quality Parameters

At commercial maturation stage (corresponding approximately to 80% of the total fruit surface colored) strawberry fruits were harvested and quality parameters were analyzed. The fruits color was recorded according to the CIELAB scale and no significant differences were highlighted depending on the Se fertilization as compared to control fruits (**Supplementary Table S1**). Total soluble solids (TSS), expressed as degrees Brix grade (°Bx), have been affected significantly by the treatment; in fact, a 30% increase in TSS has been observed in strawberries treated with 100 μM Se respect to the control and 10 μM Se-treated plants (**Table 3**). Titratable acidity showed no significant difference between treatments (**Table 3**). The sweetness index [expressed as the ratio of TSS and the acidity (Sturm et al., 2003)] of strawberry fruits harvested from control plants resulted 4.94, whereas plants supplied with 10 μM Se exhibited an index of 5.27 and those treated with 100 μM Se had an index of 6.46 (**Table 3**). On the other hand, firmness was not affected by Se supplementation (**Table 3**).

**Table 3:** Total soluble solids (°Bx), titratable acidity expressed as % citric acids, sweetness index and firmness of strawberries grown in a full nutrient (control) and a nutrient solution either supplied with 10 or 100 μM Se.

	Control	10 μM Se	100 μM Se	*P*
Total soluble solids (°Bx)	5.88 ± 0.24^b^	5.64 ± 0.21^b^	7.77 ± 0.36^a^	**0.038**
Titratable acidity (% citric acid)	0.89 ± 0.00^ns^	0.90 ± 0.04^ns^	0.96 ± 0.02^ns^	0.188
Sweetness index	4.94 ± 0.14^b^	5.27 ± 0.20^b^	6.46 ± 0.53^a^	**0.043**
Firmness (N)	1.57 ± 0.06^ns^	1.62 ± 0.06^ns^	1.60 ± 0.06^ns^	0.864

*mean ± SE; letters following the means indicate significant differences; ns, not significant. Bold *p*-values highlight statistical significance.*

### Sugars, Organic Acids and Phenolic Compounds Content of Strawberries

**FIGURE 1:**
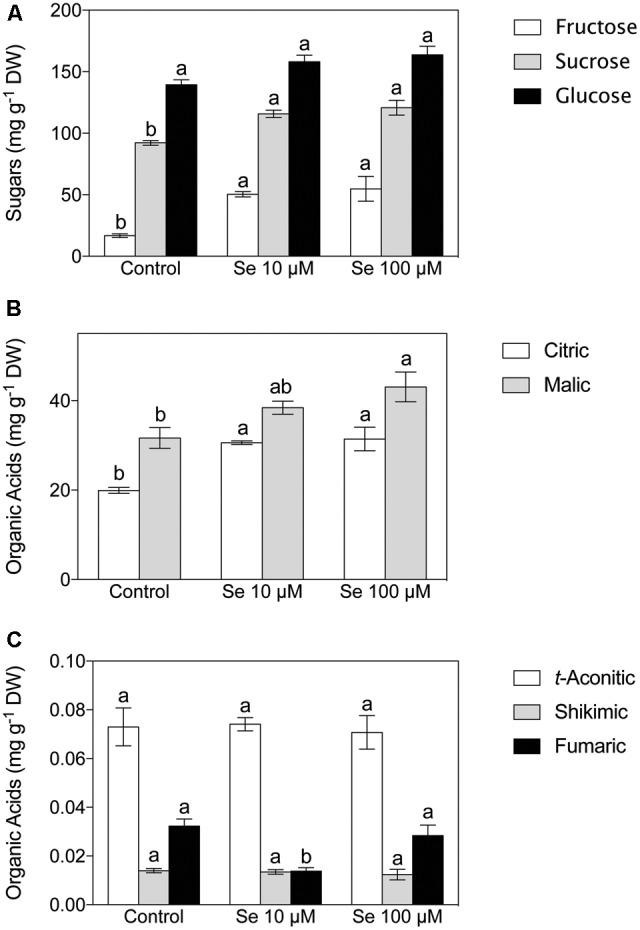
Sugar **(A)** and Organic Acid **(B,C)** concentration of strawberry fruit grown in a full nutrient (control) or in a nutrient solution either supplied with 10 μM Se (Se 10 μM) or 100 μM (Se 100 μM). Data are reported as means ± SE, *n* = 3. The statistical significance was tested by means of ANOVA with Tukey post-test. Different letters indicate statistically different values (*P* ≤ 0.05).

Fructose, sucrose and glucose were the predominant sugars detected in the extract of strawberry fruits (**Figure 1A**). The content of soluble sugars was significantly affected by the Se treatment imposed; in fact, the concentration of both fructose and sucrose resulted significantly higher in the plants supplied with 10 and 100 μM Se respect to the control plants; on the other hand, glucose showed no significant differences (**Figure 1A**).

**FIGURE 2:**
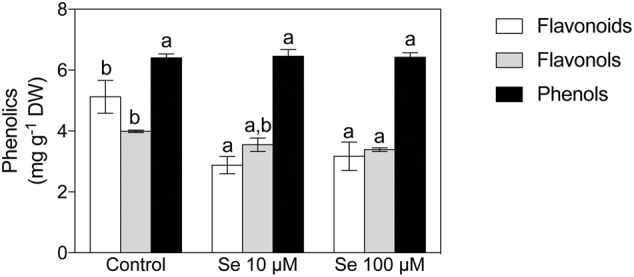
Total phenols, total flavonoids and total flavonols concentration in strawberry fruits grown in a full nutrient (control) and a nutrient solution either supplied with 10 or 100 μM Se. Data are reported in the graph as means ± SE, *n* = 3. The statistical significance was tested by means of ANOVA with Tukey post-test. Different letters indicate statistically different values (*P* ≤ 0.05).

Concerning organic acids, citric, malic, *t*-aconitic, shikimic and fumaric acid were the predominant compounds detected in the strawberry fruit extracts (**Figures 1B,C**). In particular, citric and malic acids were the two most abundant and they were typically present at concentrations 1000-fold higher than the other acids detected (**Figures 1B,C**). Total phenol content was not affected by Se treatment (**Figure 2**), however, the concentration of flavonoids and flavonols, which are subclasses of phenolic compounds, decreased significantly with increasing Se supplementation (**Figure 2**).

### Metabolomic Profile of Strawberry Fruits

**FIGURE 3:**
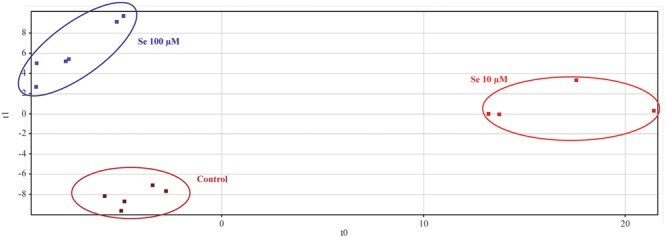
Partial Least Squares Discriminant Analysis (PLS-DA) carried out on strawberry fruit samples produced by plants grown in a full nutrient (control) and a nutrient solution either supplied with 10 or 100 μM Se.

Metabolic profiling of strawberry fruits has been carried out by an untargeted analysis through a hybrid quadrupole-time-of-flight mass spectrometer coupled to an UHPLC chromatographic system. Metabolites were annotated by comparison with Plant Metabolic Network (PMN) database^3^. The Partial Least Square Discriminant Analysis (PLS-DA), carried out on the metabolites identified, showed a clear separation (overall validation accuracy after validation and model training = 100%) of the samples according to the Se concentration applied to the nutrient solution, thus demonstrating that the metabolome of fruits was differentially affected by the biofortification practice (**Figure 3**).

Differential compounds, as highlighted by the Volcano plot analysis, are given in **Table 4**. As reported, extreme variations (given as fold-change = 16 and *p* = 0) could be observed for a large number of metabolites; the alteration of the secondary metabolites occurred particularly in cytokinins and amino acids (**Table 4**). However, also other classes, such as alkaloids, aldoximes and brassinosteroids, have been influenced by the Se treatment. Among the cytokinins, dihydrozeatin was up accumulated in both 10 and 100 μM Se treated plants respect to the control fruits.

**Table 4:** Volcano analysis (moderated *t*-test, *p* ≤ 0.05, fold change cut off = 2) of strawberry metabolites identified using the database PlantCyc.

Compound	p (Corr)	Fold-change	Regulation
**10 μM Se versus control**			
Hexadecanedioate	0	16	Up
Afrormosin-7-*O*-glucoside	0	16	Up
4α-carboxy-5α-cholesta-8.24-dien-3β-ol	0.033	3.7	Down
6-methylthiohexylhydroximoyl-glutathione	0	16	Up
Typhasterol	0	16	Up
3-hydroxy-2-oxo-indole-3-acetate	0	16	Up
Glycyrrhetaldehyde	0.006	4.9	Down
4α-carboxy,4β,14α-dimethyl-9β-19-cyclo-5α-cholest-24-en-3β-ol	0.040	3.7	Down
(*-*)-(*R*)-carvone	0	16	Up
1-palmitoylglycerol 3-phosphate	0	16	Up
(+)-copalyl diphosphate	0	16	Up
*S*-(indolylmethylthiohydroximoyl)-L-cysteine	0	2.9	Down
Apigenin 7-*O*-glucoside	0	3.7	Down
*p*-aminobenzoyl glutamate	0	16	Up
7-*O*-methylvitexin 2″-*O*-β-rhamnoside	0	16	Up
3,4-dihydroxymandelonitrile-β-glucoside	0	16	Down
*N*-formyl-D-kynurenine	0	16	Down
**100 μM Se versus control**			
Dihydrozeatin	0	16	Up
(13E)-11α-hydroxy-9,15-dioxoprost-13-enoate	0	16	Up
(3E)-phytochromobilin	0.042	3.1	Up
4-(1-methyl-5-hydroxy-2-pyrrolidinyl)-3-oxobutanoate methyl ester	0	4.8	Up
D-octopine	0	16	Up
Phenylalanine	0	16	Up
N^6^-dimethylallyladenine	0	16	Up
Gramine	0	4.2	Up
Pamidronate	0	16	Up
Neryl cation	0	16	Up
kaempferide 3-*O*-glucopyranosyl-(1-2)-*O*-rhamnoside	0	16	Up
Indolylmethylthiohydroximate	0	16	Down
3,4-dihydroxymandelonitrile β-glucoside	0	16	Down
*N*-formyl-D-kynurenine	0	16	Down

*Differential metabolites are presented as Bonferroni corrected p, fold-change (absolute values, in either 10 or 100 μM Se versus control) and regulation. According to the software Mass Profiler Professional 12.06 used, *p-*values of 0 and fold-change values of 16 denote extremely high significance or fold-change, respectively.*

Within the amino acids metabolic pathways, Se treated strawberry fruits showed an over accumulation of phenylalanine, which belongs to the phenylalanine metabolism and it is an intermediate for the biosynthesis of *N*-acetyl-phenylalanine; furthermore, D-octopine, involved in the arginine and proline metabolism, was also up accumulated in Se biofortified fruits. On the other hand, the down accumulation of both indolylmethylthiohydroximate and *N*-formyl-D-kynurenine in Se treated plants suggests that Se enrichment affected the glucosinolate biosynthesis and the aromatic amino acid synthesis. Selenium treatments induced also an increase in the content of alkaloid such as gramine that might have a role in plant defense against insects (Wippich and Wink, 1985; Corcuera, 1993). Moreover, nitrogen-containing bisphosphonate, pamidronate has been upregulated in Se treated plants.

Afrormosin-7-*O*-glucoside, an isoflavone belonging to the family of flavonoids, was specifically down accumulated in 10 μM Se treated plants, whilst no significant alteration was detected in 100 μM Se treated fruits. Furthermore, the brassinosteroid typhasterol, which is an intermediate metabolite in the brassinosteroid and acts as plant hormone with plant growth-promoting activity (Takatsuto, 1986; Kauschmann et al., 1996), was down accumulated in 10 μM Se treated strawberries. The treatment with 10 μM Se induced also a down accumulation of 3,4-dihydroxymandelonitrile-β-glucoside, a member of the cyanogenic glycoside group, (-)-(*R*)-carvone, a monoterpenoid commonly found in essential oils, and (+)-copalyl diphosphate, involved in the biosynthesis of abietate, palustric acid or levopimaric acid. Interestingly, the (3E)-phytochromobilin, which is the chromophor group of the phytochrome involved in the perception of the red and far-red light, is up accumulated in strawberry fruits treated with 100 μM Se. Experimental evidence shows that the perception of light and the subsequent signaling are responsible for an increased biosynthesis and accumulation of flavonoids (Zoratti et al., 2014).

In order to gain further details on metabolites profile, particularly from a nutritional point of view, the Phenol-Explorer (Rothwell et al., 2013) database, encompassing only phenolic compounds occurring in plants, was also used. Flavonoids, in particular anthocyanins, and polyphenols were the predominant compound classes found in strawberry fruits (**Table 5**). Most of these metabolites have been up accumulated in both 10 and 100 μM Se treated plants compared to control plants. Only pelargonidin 3-*O*-sambubioside, an anthocyanin, has been down accumulated in strawberries supplied with 100 μM Se respect to control plants. On the other hand, delphinidin 3,5-*O*-diglucoside, another member of the anthocyanin class, has been found to be up accumulated in plants supplied with 10 μM Se. Among the polyphenols, 3,4-DHPEA-EDA, specifically a tyrosol, was the compound with the highest positive fold change of about 2000 in 100 μM Se treated plants compared to control plants. However, in strawberries supplied with only 10 μM Se the up-regulation occurred at 15 times fold-change. The second most abundant compound was coumestrol, which is an end product of the isoflavonoid biosynthesis or phenylpropanoid biosynthesis. It has been up accumulated approximately 1300 times in strawberries supplied with Se, independently from the concentration. The phenolics induced by supplementation were the same at either 10 or 100 μM Se, suggesting that the effect was specifically related to Se. Interestingly, when the highest and the lowest concentrations were compared, 100 μM Se treatment was slightly more effective in inducing the accumulation of anthocyanins, with delphinidin 3,5-*O*-diglucoside and pelargonidin 3-*O*-sambubioside having a negative fold-change of 6 and 2 respectively in 100 μM Se.

**Table 5:** Volcano analysis (moderated *t*-test, *p* ≤ 0.05, fold change cut off = 2) of strawberry phenolic compounds.

Compound	p (Corr)	Fold-change	Regulation
**10 μM Se versus control**			
Delphinidin 3,5-*O*-diglucoside	0	3.9	up
Coumestrol	0	1367.6	up
3,4-DHPEA-EDA	0	15.0	up
Diosmin	0	16	up
Peonidin 3-*O*-(6”-*p*-coumaroyl-glucoside)	0	16	up
Petunidin 3-*O*-(6”-*p*-coumaroyl-glucoside)	0	2.6	up
**100 μM Se versus control**			
Coumestrol	0	1208.8	up
3,4-DHPEA-EDA	0	1956.9	up
Diosmin	0	16	up
Peonidin 3-*O*-(6”-*p*-coumaroyl-glucoside)	0	16	up
Pelargonidin 3-*O*-sambubioside	0	4.9	down

*Differential phenolics are presented as Bonferroni corrected p, fold-change (absolute values, in either 10 or 100 μM Se versus control) and regulation. According to the software Mass Profiler Professional 12.06 used, *p-*values of 0 and fold-change values of 16 denote extremely high significance or fold-change, respectively.*

Naïve Bayesian biomarker discovery confirmed the involvement of phenolic compounds in response to Se supplementation, as coumestrol, conjugated ferulic acid and glycosylated anthocyanins [malvidin 3-*O*-(6″-*p*-coumaroyl)-glucoside and pelargonidin 3-*O*-sambubioside].

## Discussion

Nutrients derived from plant-based foods are an asset for an appropriate human nutrition. However, edible crops often lack many essential nutrients (or their concentration is not enough to assure an equilibrate growth); fertilization practices that are traditionally adopted in agriculture can, on the other hand, only partially improve the nutritional value of some foods. For these reasons, in recent years, several studies have been aimed at setting up agricultural practices in order to develop functional foods fortified for selected nutraceuticals, such as mineral micronutrients, antioxidants and vitamins (Hirschi, 2009). Selenium for instance, being an essential micronutrient for both human and animals (Malagoli et al., 2015), is gaining more and more interest due to its influence on the quality of agricultural products. In fact, several studies described the biofortification potential to obtain Se-enriched rice, edible sprouts, mushrooms, carrots, potatoes, chickpeas, corn salad and tomatoes (Kápolna et al., 2009; Funes-Collado et al., 2013; Schiavon et al., 2013; Wang et al., 2013; Poblaciones et al., 2014; Niedzielski et al., 2015; Tomasi et al., 2015).

The present research had the aim of investigating the feasibility of Se biofortification of fruit crops using hydroponically cultivated strawberry plants. In herbaceous species (e.g., ryegrass, lettuce, potato and tomato), the application of low doses of Se (e.g., 10 μM) had growth-promoting effects, whilst higher concentrations induced toxicity symptoms in plants (Hawrylak-Nowak, 2009; Pilon-Smits et al., 2009; Schiavon et al., 2013). For these reasons, the healthy status of strawberry plants was assessed evaluating the biometric parameters, such as biomass allocation and leaf area (**Table 1**): low Se concentrations (10 μM) did not affect the growth of plants as compared with controls, whereas high Se concentrations (100 μM Se) induced a significant accumulation of shoot biomass (+22%) and a wider leaf area (+17%). Furthermore, the supplementation of Se in the nutrient solution did not negatively influence the fertility of strawberries (i.e., fruit-set) and did not reduce the fruit yield, as also observed in tomatoes treated with lower Se concentrations (Lee et al., 2007; Pezzarossa et al., 2014; Castillo-Godina et al., 2016). It has been suggested that the effects of Se on plants could mainly depend on its tissues concentrations (Hamilton, 2004) and, most importantly, on the sulfur (S)-to-Se ratio (White et al., 2004; El Kassis et al., 2007; Barberon et al., 2008). In particular, it was observed that low [S]/[Se] ratios correlate with the appearance of toxicity symptoms (White et al., 2004). In our study, the application of Se to the nutrient solution resulted in a proportional increase in Se concentration in the plant tissues (i.e., roots and leaves) as compared to control plants, whilst S concentration was enhanced limitedly to the shoots of strawberries fertilized with the highest Se concentration (100 μM Se, **Table 2**). Ten μM Se-treated strawberry plants showed a [S]/[Se] ratio approximately 10-fold higher than plants supplied with 100 μM Se (170 vs. 18, respectively). Although low ratios like 20 were shown to correlate with the manifestation of Se stress in tomato plants (Schiavon et al., 2013), our strawberry plants did not exhibit toxicity symptoms. Our results might suggest that strawberries have a different tolerance to Se than other herbaceous species, most likely due to a different Se uptake efficiency, which is known to be dependent on the plant species (Rayman, 2008; Zhu et al., 2009; Thiry et al., 2012).

Besides Se, the concentrations of other essential elements are also fundamental parameters to assess the effectiveness of the biofortification approach. To the very best of our knowledge, only few studies of Se-enriched vegetables considered the dynamics of micro- and macronutrients other than S and were focused almost exclusively on shoots; yet these works mainly highlighted species-specific behaviors of mineral elements (Hawrylak-Nowak, 2009; Filek et al., 2010; Ramos et al., 2011; Saffaryadzi et al., 2012; Smoleń et al., 2014; Hawrylak-Nowak et al., 2015). Indeed, the alteration of the composition of the nutrient solution (i.e., supplementation with Se) might induce an altered uptake of other mineral nutrients (Pii et al., 2015; Valentinuzzi et al., 2015a). Overall, Se treatments did not induce dramatic modifications of the ionomic profile of both roots and shoots. For instance, roots of 100 μM Se-treated plants exhibited significantly higher K, Mg and Ca concentrations than the roots of control plants (**Table 2**).

Fruits produced by plants cultivated in Se-fortified nutrient solutions showed a significant accumulation of Se (**Table 2**). The recommended Se daily intake for humans is estimated to lay between of 55–200 μg (USDA, 2012), thus an average serving of 10 μM Se biofortified strawberries (∼150 g) would supply consumers with 60 μg of the microelement. On the other hand, the consumption of a serving of 100 μM Se biofortified strawberries would supply consumers with approximately 600 μg day^-1^, which exceeds the toxic limit for humans (400 μg day^-1^) (USDA, 2012). Beside the increase in the Se concentrations, the only effects observed in the mineral element content of fruit was a reduction in the concentration of Ca, which might represent an important factor determining quality. In fact, fruits with low Ca content are sensitive to many physiological and pathological disorders and generally have a short shelf-life (Wójcik and Lewandowski, 2003). Nevertheless, in the present case, the firmness of strawberries was not affected by Se fertilization (**Table 3**).

Dissimilarities in the nutrient profiles among plant organs (e.g., roots vs. fruits) are most likely ascribable to their different nutrient requirements based on their developmental stage (i.e., flowering, fruit-set).

Furthermore, soluble solids content and the sweetness index were also enhanced in Se-biofortified fruits (**Table 3**), suggesting that Se might also have a positive influence on the fruit taste. According to previous works, strawberry fruits with a sweetness index of 7 where considered as sweet, whilst indexes equal to 6 indicated an acid flavor, therefore these data suggested that Se enrichment might have a positive influence on fruit ripening and sugar metabolism (Wozniak et al., 1997). Indeed, recent studies run on *Camellia sinensis* leaves have highlighted a positive correlation between the concentration of Se and both the soluble sugars and the sweetness index (Hu et al., 2003; Zhao et al., 2016).

The variation in Se availability to plants has been often related to an alteration of the S uptake, thus inducing changes in the synthesis of S-secondary metabolites that have been characterized for their very high nutritional value (Malagoli et al., 2015). The metabolomic analysis of strawberries showed that both intermediates of the glucosinolate (GLS) biosynthesis (i.e., *N*-formyl-D-kynurenine and indolylmethylthiohydroxiamte) and the amino acid S-(indolylmethylthiohydroximoyl)-L-cysteine, which is involved in the metabolism of tryptophan, are down accumulated in Se-treated plants (**Table 4**); such a down accumulation has already been observed in other species (Ávila et al., 2013, 2014). Even though no significant variation of the S concentration was observed in fruits, these findings might suggest the occurrence of a hidden competition between S and Se, which prevents plants from correctly assimilate the macronutrient S. In addition, it was also shown that Se treatments can also induce the upregulation of genes involved in the GLS catabolism (Van Hoewyk et al., 2008).

Several studies also highlighted contrasting effects of Se fertilization on the concentration of total phenols, either positive or negative, depending on the plant species and the concentration of Se applied (Finley et al., 2005; Robbins et al., 2005; Ríos et al., 2008; Schiavon et al., 2013). In the case of strawberries, the concentration of total phenolic compounds was not affected by the biofortification, albeit the total flavonoids concentration was statistically decreased by Se treatments (**Figure 2**). In spite of this evidence, Se caused an upregulation of the phenylpropanoid biosynthetic pathway leading to the accumulation of specific metabolites; these metabolites belong to the flavonoid and polyphenol classes (i.e., Coumestrol, Peonidin 3-*O*-(6″-*p*-coumaroyl-glucoside) and Diosmin) and play a central role in determining the organoleptic features and the antioxidant capacity of strawberry fruits (**Table 5**). Interestingly, the metabolomics profile of Se-biofortified strawberries also highlighted the up accumulation of dihydrozeatin, an intermediate in the zeatin biosynthesis. Zeatin is a cytokinin known to be involved in controlling the cell division (Blanco et al., 2000) and the shoot-to-root ratio (Kaul et al., 2000; Werner et al., 2003). The up accumulation of this intermediate further supports the effects observed on the biometric parameters (**Table 1**). In addition, cytokinins have been recently demonstrated to be related to the ripening process in *Vitis vinifera* (Böttcher et al., 2015) and with the biosynthesis of flavonoids in *Arabidopsis thaliana* (Das et al., 2012).

In spite of these pieces of evidence, the metabolomic analysis did not highlight a statistically significant accumulation of Se-containing metabolites in the biofortified strawberries. Indeed, it has been shown that Se can be accumulated as S-analog amino acids, as for instance selenocysteine and selenomethionine, which can be used in proteins synthesis; however, in some cases, the incorporation of Se-amino acids in proteins can be avoided by diverting Se to non-proteinogenic aminoacids, namely methylselenocysteine (MeSeCys), γ-glutamyl-MeSeCys and/or selenocystationine. Furthermore, Se could also be volatilized from plants in the forms of dimethylselenide or dimethyldiselenide (Malagoli et al., 2015).

## Conclusion

Based on our observations, strawberry plants seem a good target for Se biofortification to increase the human intake of this essential micronutrient without impairing growth and yield parameters. Indeed, 10 μM Se-treated strawberries would supply consumers with 60 μg Se per day considering a serving of approximately 150 g of fresh strawberries. Moreover, since 60 μg correspond to the lower limit of the recommended daily intake, it could be of greater benefit to even increase the Se enrichment using nutrient solutions supplemented with Se concentration higher than 10 μM; contrarily, 100 μM Se supplementation resulted in potentially harmful fruits for human health. The untargeted metabolic profiling has further highlighted that Se induced the up regulation of several metabolic pathways, especially those involved in the synthesis of antioxidant compounds. Selenium enrichments could thus play a pivotal role in triggering metabolic pathways leading to an increase in health beneficial compounds.

## Author Contributions

Designing and performing the experiments: TM, RT, FV, and SC. Data analyses: YP, LL, TM, and RT. Critical discussion of the data: TM, RT, FV, LL, CN, PS, MS, YP, and SC. Paper preparation: YP, TM, and SC. Research coordination: TM and SC.

## Conflict of Interest Statement

The authors declare that the research was conducted in the absence of any commercial or financial relationships that could be construed as a potential conflict of interest.

## Footnotes

1 http://www.plantcyc.org

2 http://cosmos-fp7.eu/msi

3 www.plantcyc.org

## Supplementary Material

FIGURE S1 SPAD index values of leaves measured in control, Se 10 and Se 100 μM strawberry plants at the end of the production period; error bars indicate the standard error (SE), (*n* = 6).

TABLE S1 Skin color parameters (L^∗^, a^∗^ and b^∗^) evaluated for strawberry fruits grown either in complete nutrient solution (control), either in nutrient solution supplied with 10 μM Se or in nutrient solution supplied with 100 μM Se.

## References

[B1] Atanassova M. Georgieva S. Ivancheva K. (2011). Total phenolic and total flavonoid contents, antioxidant capacity and biological contaminants in medicinal herbs. *J. Univ. Chem. Technol. Metall.* 46 81–88.

[B2] Ávila F. W. Faquin V. Yang Y. Ramos S. J. Guilherme L. R. G. Thannhauser T. W. (2013). Assessment of the anticancer compounds Se-Methylselenocysteine and Glucosinolates in Se-biofortified broccoli (*Brassica oleracea* L. var. *italica*) sprouts and florets. *J. Agric. Food Chem.* 61 6216–6223. 10.1021/jf4016834 23763668

[B3] Ávila F. W. Yang Y. Faquin V. Ramos S. J. Guilherme L. R. G. Thannhauser T. W. (2014). Impact of selenium supply on Se-methylselenocysteine and glucosinolate accumulation in selenium-biofortified Brassica sprouts. *Food Chem.* 165 578–586. 10.1016/j.foodchem.2014.05.134 25038715

[B4] Barberon M. Berthomieu P. Clairotte M. Shibagaki N. Davidian J.-C. Gosti F. (2008). Unequal functional redundancy between the two *Arabidopsis thaliana* high-affinity sulphate transporters *SULTR1*;*1* and *SULTR1*;*2*. *New Phytol.* 180 608–619. 10.1111/j.1469-8137.2008.02604.x 18761637

[B5] Blanco M. H. Quintana M. del C. Hernández L. (2000). Determination of Dihydrozeatin and Dihydrozeatin riboside by cathodic stripping voltammetry. *Electroanalysis* 12 147–154. 10.1002/(SICI)1521-4109(200002)12:2<147::AID-ELAN147>3.0.CO;2-F

[B6] Böttcher C. Burbidge C. A. Boss P. K. Davies C. (2015). Changes in transcription of cytokinin metabolism and signalling genes in grape (*Vitis vinifera* L.) berries are associated with the ripening-related increase in isopentenyladenine. *BMC Plant Biol.* 15:223. 10.1186/s12870-015-0611-5 26377914 PMC4573921

[B7] Brown T. A. Shrift A. (1982). Selenium: toxicity and tolerance in higher plants. *Biol. Rev.* 57 59–84. 10.1111/j.1469-185X.1982.tb00364.x

[B8] Brummell D. A. Watson L. M. Pathirana R. Joyce N. I. West P. J. Hunter D. A. (2011). Biofortification of tomato (*Solanum lycopersicum*) fruit with the anticancer compound methylselenocysteine using a selenocysteine methyltransferase from a selenium hyperaccumulator. *J. Agric. Food Chem.* 59 10987–10994. 10.1021/jf202583f 21942920

[B9] Castillo-Godina R. G. Foroughbakhch-Pournavab R. Benavides-Mendoza A. (2016). Effect of selenium on elemental concentration and antioxidant enzymatic activity of tomato plants. *J. Agric. Sci. Technol.* 18 233–244.

[B10] Castro I. Goncalves O. Teixeira J. A. Vicentesc A. A. (2002). Comparative study of Selva and Camarosa strawberries for the commercial market. *J. Food Sci.* 67 2132–2137. 10.1111/j.1365-2621.2002.tb09515.x

[B11] Chen L. Yang F. Xu J. Hu Y. Hu Q. Zhang Y. (2002). Determination of Selenium concentration of rice in China and effect of fertilization of Selenite and Selenate on Selenium content of rice. *J. Agric. Food Chem.* 50 5128–5130. 10.1021/jf0201374 12188618

[B12] Corcuera L. J. (1993). Biochemical basis for the resistance of barley to aphids. *Phytochemistry* 33 741–747. 10.1016/0031-9422(93)85267-U

[B13] Das P. K. Shin D. H. Choi S.-B. Yoo S.-D. Choi G. Park Y.-I. (2012). Cytokinins enhance sugar-induced anthocyanin biosynthesis in *Arabidopsis*. *Mol. Cells* 34 93–101. 10.1007/s10059-012-0114-2 22699753 PMC3887782

[B14] Dennert G. Zwahlen M. Brinkman M. Vinceti M. Zeegers M. P. A. Horneber M. (2011). Selenium for preventing cancer. *Cochrane Database Syst. Rev.* 10.1002/14651858.CD005195.pub2 24683040 PMC4441528

[B15] DeTar R. A. Alford ÉR. Pilon-Smits E. A. H. (2015). Molybdenum accumulation, tolerance and molybdenum–selenium–sulfur interactions in *Astragalus* selenium hyperaccumulator and nonaccumulator species. *J. Plant Physiol.* 183 32–40. 10.1016/j.jplph.2015.05.009 26074355

[B16] El Kassis E. Cathala N. Rouached H. Fourcroy P. Berthomieu P. Terry N. (2007). Characterization of a selenate-resistant *Arabidopsis* Mutant. Root growth as a potential target for selenate toxicity. *Plant Physiol.* 143 1231–1241. 10.1104/pp.106.091462 17208959 PMC1820920

[B17] Filek M. Zembala M. Kornaś A. Walas S. Mrowiec H. Hartikainen H. (2010). The uptake and translocation of macro- and microelements in rape and wheat seedlings as affected by selenium supply level. *Plant Soil* 336 303–312. 10.1007/s11104-010-0481-4

[B18] Finley J. W. Sigrid-Keck A. Robbins R. J. Hintze K. J. (2005). Selenium enrichment of broccoli: interactions between Selenium and secondary plant compounds. *J. Nutr.* 135 1236–1238. 15867310 10.1093/jn/135.5.1236

[B19] Folin O. Ciocalteu V. (1927). On tyrosine and tryptophane determinations in proteins. *J. Biol. Chem.* 27 627–650.

[B20] Fordyce F. (2007). Selenium geochemistry and health. *AMBIO* 36 94–97. 10.1579/0044-7447(2007)36[94:SGAH]2.0.CO;2 17408199

[B21] Funes-Collado V. Morell-Garcia A. Rubio R. López-Sánchez J. F. (2013). Study of selenocompounds from selenium-enriched culture of edible sprouts. *Food Chem.* 141 3738–3743. 10.1016/j.foodchem.2013.06.090 23993543

[B22] Galeas M. L. Klamper E. M. Bennett L. E. Freeman J. L. Kondratieff B. C. Quinn C. F. (2008). Selenium hyperaccumulation reduces plant arthropod loads in the field. *New Phytol.* 177 715–724. 10.1111/j.1469-8137.2007.02285.x 18028291

[B23] Giampieri F. Tulipani S. Alvarez-Suarez J. M. Quiles J. L. Mezzetti B. Battino M. (2012). The strawberry: composition, nutritional quality, and impact on human health. *Nutrition* 28 9–19. 10.1016/j.nut.2011.08.009 22153122

[B24] Halvorsen B. L. Holte K. Myhrstad M. C. W. Barikmo I. Hvattum E. Remberg S. F. (2002). A systematic screening of total antioxidants in dietary plants. *J. Nutr.* 132 461–471. 11880572 10.1093/jn/132.3.461

[B25] Hamilton S. J. (2004). Review of selenium toxicity in the aquatic food chain. *Sci. Total Environ.* 326 1–31. 10.1016/j.scitotenv.2004.01.019 15142762

[B26] Hartikainen H. (2005). Biogeochemistry of selenium and its impact on food chain quality and human health. *J. Trace Elem. Med. Biol.* 18 309–318. 10.1016/j.jtemb.2005.02.009 16028492

[B27] Hawrylak-Nowak B. (2009). Beneficial effects of exogenous Selenium in cucumber seedlings subjected to salt stress. *Biol. Trace Elem. Res.* 132 259–269. 10.1007/s12011-009-8402-1 19434374

[B28] Hawrylak-Nowak B. Matraszek R. Pogorzelec M. (2015). The dual effects of two inorganic selenium forms on the growth, selected physiological parameters and macronutrients accumulation in cucumber plants. *Acta Physiol. Plant.* 37 41 10.1007/s11738-015-1788-9

[B29] Hernández-Castro E. Trejo-Téllez L. Gómez-Merino F. Rodríguez-Mendoza M. Sánchez-García P. Robledo-Paz A. (2015). Bioaccumulation of iron, selenium, nitrate, and proteins in chard shoots. *J. Soil Sci. Plant Nutr.* 15 694–710. 10.4067/S0718-95162015005000047

[B30] Hirschi K. D. (2009). Nutrient biofortification of food crops. *Annu. Rev. Nutr.* 29 401–421. 10.1146/annurev-nutr-080508-141143 19400753

[B31] Hu Q. Xu J. Pang G. (2003). Effect of Selenium on the yield and quality of green tea leaves harvested in early spring. *J. Agric. Food Chem.* 51 3379–3381. 10.1021/JF0341417 12744670

[B32] Kabata-Pendias A. (2010). *Trace Elements in Soils and Plants* Fourth Edn. Boca Raton, FL: CRC Press 10.1201/b10158-25

[B33] Kápolna E. Hillestrøm P. R. Laursen K. H. Husted S. Larsen E. H. (2009). Effect of foliar application of selenium on its uptake and speciation in carrot. *Food Chem.* 115 1357–1363. 10.1016/j.foodchem.2009.01.054

[B34] Kaul S. Koo H. L. Jenkins J. Rizzo M. Rooney T. Tallon L. J. (2000). Analysis of the genome sequence of the flowering plant *Arabidopsis thaliana*. *Nature* 408 796–815. 10.1038/35048692 11130711

[B35] Kauschmann A. Jessop A. Koncz C. Szekeres M. Willmitzer L. Altmann T. (1996). Genetic evidence for an essential role of brassinosteroids in plant development. *Plant J.* 9 701–713. 10.1046/j.1365-313X.1996.9050701.x

[B36] Lee G.-J. Kang B.-K. Kim T.-I. Kim T.-J. Kim J.-H. (2007). Effects of different selenium concentrations of the nutrient solution on the growth and quality of tomato fruit in hydroponics. *Acta Hortic.* 761 443–448. 10.17660/ActaHortic.2007.761.61

[B37] Lucini L. Rouphael Y. Cardarelli M. Canaguier R. Kumar P. Colla G. (2015). The effect of a plant-derived biostimulant on metabolic profiling and crop performance of lettuce grown under saline conditions. *Sci. Hortic.* 182 124–133. 10.1016/j.scienta.2014.11.022

[B38] Lyons G. H. Stangoulis J. C. R. Graham R. D. (2004). Exploiting micronutrient interaction to optimize biofortification programs: the case for inclusion of Selenium and Iodine in the HarvestPlus Program. *Nutr. Rev.* 62 247–252. 10.1111/j.1753-4887.2004.tb00047.x 15291398

[B39] Malagoli M. Schiavon M. Dall’Acqua S. Pilon-Smits E. A. H. (2015). Effects of selenium biofortification on crop nutritional quality. *Front. Plant Sci.* 6:280. 10.3389/fpls.2015.00280 25954299 PMC4404738

[B40] Marschner P. (2011). *Marschner’s Mineral Nutrition of Higher Plants* 3rd Edn. London: Academic Press.

[B41] Miliauskas G. Venskutonis P. R. van Beek T. A. (2004). Screening of radical scavenging activity of some medicinal and aromatic plant extracts. *Food Chem.* 85 231–237. 10.3390/antiox2010011 26787619 PMC4665401

[B42] Niedzielski P. Mleczek M. Siwulski M. Rzymski P. Gąsecka M. Kozak L. (2015). Supplementation of cultivated mushroom species with selenium: bioaccumulation and speciation study. *Eur. Food Res. Technol.* 241 419–426. 10.1007/s00217-015-2474-2

[B43] Pezzarossa B. Rosellini I. Borghesi E. Tonutti P. Malorgio F. (2014). Effects of Se-enrichment on yield, fruit composition and ripening of tomato (*Solanum lycopersicum*) plants grown in hydroponics. *Sci. Hortic.* 165 106–110. 10.1016/j.scienta.2013.10.029

[B44] Pii Y. Cesco S. Mimmo T. (2015). Shoot ionome to predict the synergism and antagonism between nutrients as affected by substrate and physiological status. *Plant Physiol. Biochem.* 94 48–56. 10.1016/j.plaphy.2015.05.002 26004913

[B45] Pilon-Smits E. A. H. Quinn C. F. Tapken W. Malagoli M. Schiavon M. (2009). Physiological functions of beneficial elements. *Curr. Opin. Plant Biol.* 12 267–274. 10.1016/j.pbi.2009.04.009 19477676

[B46] Poblaciones M. J. Rodrigo S. Santamaria O. Chen Y. McGrath S. P. (2014). Selenium accumulation and speciation in biofortified chickpea (*Cicer arietinum* L.) under Mediterranean conditions. *J. Sci. Food Agric.* 94 1101–1106. 10.1002/jsfa.6372 23983062

[B47] Ramos S. J. Faquin V. Almeida H. J. de Ávila F. W. Guilherme L. R. G. Bastos C. E. A. (2011). Selenato e selenito na produção, nutrição mineral e biofortificação com selênio em cultivares de alface1. *Rev. Bras. Ciênc. Solo* 35 1347–1355. 10.1590/S0100-06832011000400029

[B48] Rayman M. P. (2008). Food-chain selenium and human health: emphasis on intake. *Br. J. Nutr.* 100 254–268. 10.1017/S0007114508939830 18346308

[B49] Rayman M. P. (2012). Selenium and human health. *Lancet* 379 1256–1268. 10.1016/S0140-6736(11)61452-9 22381456

[B50] Ríos J. J. Rosales M. A. Blasco B. Cervilla L. M. Romero L. Ruiz J. M. (2008). Biofortification of Se and induction of the antioxidant capacity in lettuce plants. *Sci. Hortic.* 116 248–255. 10.1016/j.scienta.2008.01.008

[B51] Robbins R. J. Keck A.-S. Banuelos G. Finley J. W. (2005). Cultivation conditions and Selenium fertilization alter the phenolic profile, glucosinolate, and sulforaphane content of broccoli. *J. Med. Food* 8 204–214. 10.1089/jmf.2005.8.204 16117613

[B52] Rothwell J. A. Perez-Jimenez J. Neveu V. Medina-Remón A. M’Hiri N. García-Lobato P. (2013). Phenol-Explorer 3.0: a major update of the Phenol-Explorer database to incorporate data on the effects of food processing on polyphenol content. *Database* 2013:bat070. 10.1093/database/bat070 24103452 PMC3792339

[B53] Saffaryadzi A. Lahouti M. Ganjeali A. Bayat H. (2012). Impact of Selenium supplementation on growth and Selenium accumulation on spinach (*Spinacia oleracea* L.) plants. *Not. Sci. Biol.* 4 95–100.

[B54] Schiavon M. Dall’acqua S. Mietto A. Pilon-Smits E. A. H. Sambo P. Masi A. (2013). Selenium fertilization alters the chemical composition and antioxidant constituents of tomato (*Solanum lycopersicon* L.). *J. Agric. Food Chem.* 61 10542–10554. 10.1021/jf4031822 24079300

[B55] Shibagaki N. Rose A. McDermott J. P. Fujiwara T. Hayashi H. Yoneyama T. (2002). Selenate-resistant mutants of *Arabidopsis thaliana* identify *Sultr1;2*, a sulfate transporter required for efficient transport of sulfate into roots. *Plant J.* 29 475–486. 10.1046/j.0960-7412.2001.01232.x 11846880

[B56] Shinmachi F. Buchner P. Stroud J. L. Parmar S. Zhao F.-J. McGrath S. P. (2010). Influence of sulfur deficiency on the expression of specific sulfate transporters and the distribution of Sulfur, Selenium, and Molybdenum in wheat. *Plant Physiol.* 153 327–336. 10.1104/pp.110.153759 20219830 PMC2862427

[B57] Smoleń S. Kowalska I. Sady W. (2014). Assessment of biofortification with iodine and selenium of lettuce cultivated in the NFT hydroponic system. *Sci. Hortic.* 166 9–16. 10.1016/j.scienta.2013.11.011

[B58] Sors T. G. Ellis D. R. Salt D. E. (2005). Selenium uptake, translocation, assimilation and metabolic fate in plants. *Photosynth. Res.* 86 373–389. 10.1007/s11120-005-5222-9 16307305

[B59] Stoner G. D. Wang L. S. (2013). “Chemoprevention of esophageal squamous cell carcinoma with berries,” in *Natural Products in Cancer Prevention and Therapy* eds Pezzuto M. J. Suh N. (Berlin: Springer) 1–20. 10.1007/128_2012_343 22752584

[B60] Sturm K. Koron D. Stampar F. (2003). The composition of fruit of different strawberry varieties depending on maturity stage. *Food Chem.* 83 417–422. 10.1016/S0308-8146(03)00124-9

[B61] Takatsuto S. (1986). Synthesis of teasterone and typhasterol, brassinolide-related steroids with plant-growth-promoting activity. *J. Chem. Soc. Perkin Trans.* 1 1833–1836. 10.1039/p19860001833

[B62] Thavarajah D. Ruszkowski J. Vandenberg A. (2008). High potential for Selenium biofortification of lentils (*Lens culinaris* L.). *J. Agric. Food Chem.* 56 10747–10753. 10.1021/jf802307h 18954072

[B63] Thiry C. Ruttens A. De Temmerman L. Schneider Y.-J. Pussemier L. (2012). Current knowledge in species-related bioavailability of selenium in food. *Food Chem.* 130 767–784. 10.1016/j.foodchem.2011.07.102

[B64] Tomasi N. Pinton R. Gottardi S. Mimmo T. Scampicchio M. Cesco S. (2015). Selenium fortification of hydroponically grown corn salad (*Valerianella locusta*). *Crop Pasture Sci.* 66 1128–1136. 10.1071/CP14218

[B65] Tulipani S. Mezzetti B. Capocasa F. Bompadre S. Beekwilder J. de Vos C. H. R. (2008). Antioxidants, phenolic compounds, and nutritional quality of different strawberry genotypes. *J. Agric. Food Chem.* 56 696–704. 10.1021/jf0719959 18211027

[B66] USDA (2012). *USDA National Nutrient Database for Standard Reference, Release 25*. Washington, DC: U.S. Department of Agriculture, Agricultural Research Service.

[B67] Valentinuzzi F. Mason M. Scampicchio M. Andreotti C. Cesco S. Mimmo T. (2015a). Enhancement of the bioactive compound content in strawberry fruits grown under iron and phosphorus deficiency. *J. Sci. Food Agric.* 95 2088–2094. 10.1002/jsfa.6924 25244604

[B68] Valentinuzzi F. Pii Y. Vigani G. Lehmann M. Cesco S. Mimmo T. (2015b). Phosphorus and iron deficiencies induce a metabolic reprogramming and affect the exudation traits of the woody plant *Fragaria × ananassa*. *J. Exp. Bot.* 66 6483–6495. 10.1093/jxb/erv364 26188206

[B69] Van Hoewyk D. Takahashi H. Inoue E. Hess A. Tamaoki M. Pilon-Smits E. A. H. (2008). Transcriptome analyses give insights into selenium-stress responses and selenium tolerance mechanisms in *Arabidopsis*. *Physiol. Plant.* 132 236–253. 10.1111/j.1399-3054.2007.01002.x 18251864

[B70] Wang Y.-D. Wang X. Wong Y.-S. (2013). Generation of selenium-enriched rice with enhanced grain yield, selenium content and bioavailability through fertilisation with selenite. *Food Chem.* 141 2385–2393. 10.1016/j.foodchem.2013.05.095 23870972

[B71] Werner T. Motyka V. Laucou V. Smets R. Van Onckelen H. Schmülling T. (2003). Cytokinin-deficient transgenic *Arabidopsis* plants show multiple developmental alterations indicating opposite functions of cytokinins in the regulation of shoot and root meristem activity. *Plant Cell* 15 2532–2550. 10.1105/tpc.014928 14555694 PMC280559

[B72] White P. J. Bowen H. C. Parmaguru P. Fritz M. Spracklen W. P. Spiby R. E. (2004). Interactions between selenium and sulphur nutrition in *Arabidopsis thaliana*. *J. Exp. Bot.* 55 1927–1937. 10.1093/jxb/erh192 15258164

[B73] Wippich C. Wink M. (1985). Biological properties of alkaloids. Influence of quinolizidine alkaloids and gramine on the germination and development of powdery mildew, Erysiphe graminis f.sp.*hordei*. *Experientia* 41 1477–1479. 10.1007/BF01950046

[B74] Wójcik P. Lewandowski M. (2003). Effect of Calcium and Boron sprays on yield and quality of “Elsanta” strawberry. *J. Plant Nutr.* 26 671–682. 10.1081/PLN-120017674

[B75] Wozniak W. Radajewska B. Reszelska-Sieciechowicz A. Dejwor I. (1997). Sugars and acid content influence organoleptic evaluation of fruit of six strawberry cultivars from controlled cultivation. *Acta Hortic.* 439 333–336. 10.17660/ActaHortic.1997.439.52

[B76] Zayed A. M. Terry N. (1992). Selenium volatilization in broccoli as influenced by sulfate supply. *J. Plant Physiol.* 140 646–652. 10.1016/S0176-1617(11)81018-7

[B77] Zhao H. Huang J. Li Y. Song X. Luo J. Yu Z. (2016). Natural variation of selenium concentration in diverse tea plant (*Camellia sinensis*) accessions at seedling stage. *Sci. Hortic.* 198 163–169. 10.1016/j.scienta.2015.11.026

[B78] Zhu Y.-G. Pilon-Smits E. A. H. Zhao F.-J. Williams P. N. Meharg A. A. (2009). Selenium in higher plants: understanding mechanisms for biofortification and phytoremediation. *Trends Plant Sci.* 14 436–442. 10.1016/j.tplants.2009.06.006 19665422

[B79] Zoratti L. Karppinen K. Luengo Escobar A. Häggman H. Jaakola L. (2014). Light-controlled flavonoid biosynthesis in fruits. *Front. Plant Sci.* 5:534. 10.3389/fpls.2014.00534 25346743 PMC4191440

