# Supplementary figures and images for: Selenium Biofortification in Fragaria × ananassa: Implications on Strawberry Fruits Quality, Content of Bioactive Health Beneficial Compounds and Metabolomic Profile
Source: Front Plant Sci. 2017 Nov 6;8:1887. doi: 10.3389/fpls.2017.01887 (PMC5681748; doi:10.3389/fpls.2017.01887)

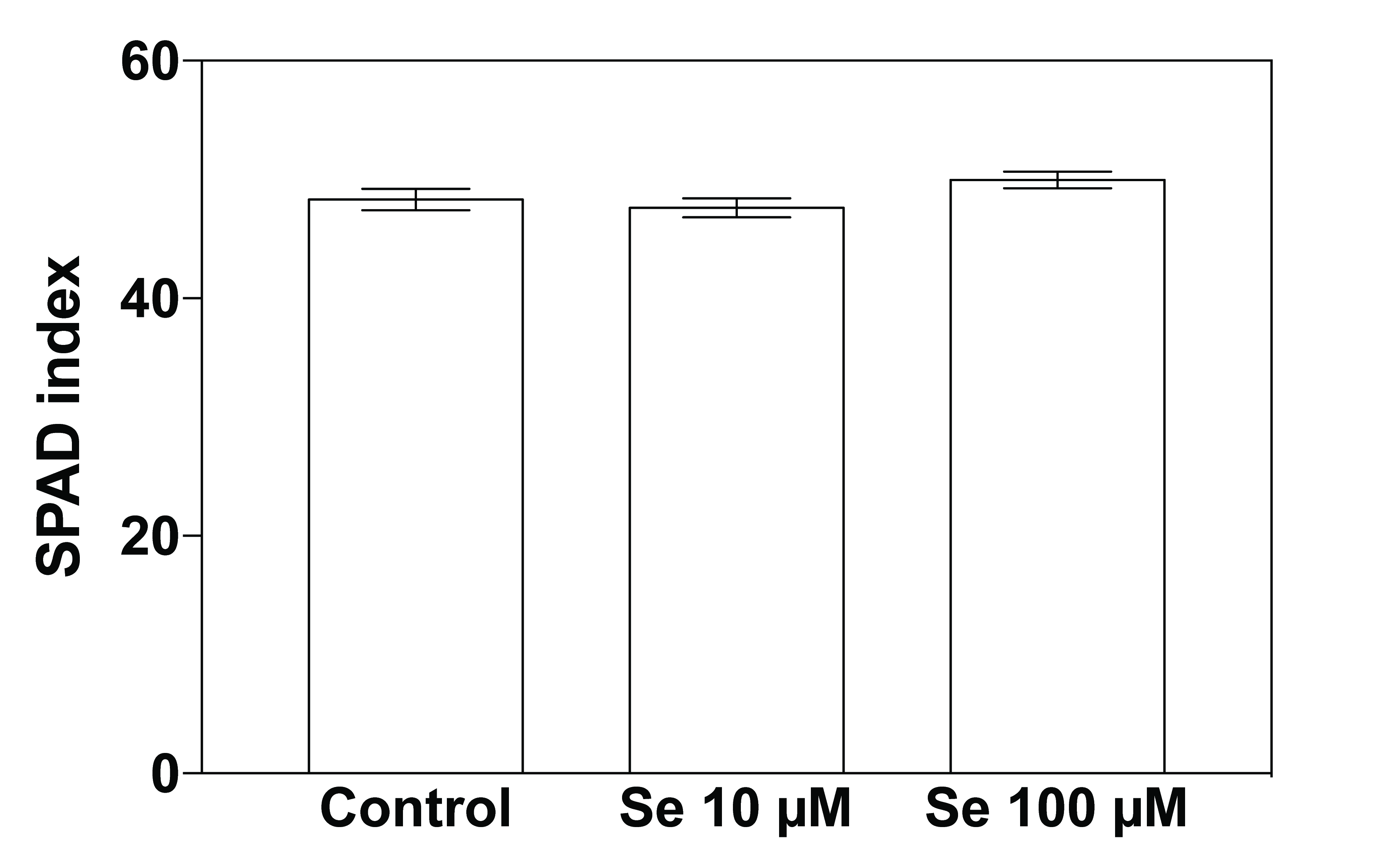

Supplement: FIGURE S1 — SPAD index values of leaves measured in control, Se 10 and Se 100 μM strawberry plants at the end of the production period; error bars indicate the standard error (SE), (n = 6). [file Image_1.TIF]
