# Supplementary material for: Selenium Biofortification in Fragaria × ananassa: Implications on Strawberry Fruits Quality, Content of Bioactive Health Beneficial Compounds and Metabolomic Profile
Source: Front Plant Sci. 2017 Nov 6;8:1887. doi: 10.3389/fpls.2017.01887 (PMC5681748; doi:10.3389/fpls.2017.01887)
Supplement: TABLE S1 — Skin color parameters (L∗, a∗ and b∗) evaluated for strawberry fruits grown either in complete nutrient solution (control), either in nutrient solution supplied with 10 μM Se or in nutrient solution supplied with 100 μM Se. [file Table_1.docx]

**Supplementary Table 1.** Skin colour parameters (L*, a* and b*) evaluated for strawberry fruits grown either in complete nutrient solution (Control), either in nutrient solution supplied with 10 μM Se or in nutrient solution supplied with 100 μM Se.

| **Treatment** | **L*** | **a*** | **b*** |
| --- | --- | --- | --- |
| Control | 42.87 ± 3.48^ns^ | 31.59 ± 1.70^ns^ | 15.18 ± 1.27^ns^ |
| 10 μM Se | 45.15 ± 4.57^ns^ | 28.81 ± 2.60^ns^ | 16.55 ± 1.86^ns^ |
| 100 μM Se | 43.38 ± 3.41^ns^ | 32.01 ± 3.33^ns^ | 16.17 ± 2.22^ns^ |
